# Oral treatment of 4-methylumbelliferone reduced perineuronal nets and improved recognition memory in mice

**DOI:** 10.1016/j.brainresbull.2022.01.011

**Published:** 2022-04

**Authors:** Jana Dubisova, Jana Svobodova Burianova, Lucie Svobodova, Pavol Makovicky, Noelia Martinez-Varea, Anda Cimpean, James W. Fawcett, Jessica C.F. Kwok, Sarka Kubinova

**Affiliations:** a Institute of Experimental Medicine of the Czech Academy of Sciences, Videnska 1083, 142 20 Prague, Czech Republic; b 2nd Medical Faculty, Charles University, V Úvalu 84, 150 06 Prague, Czech Republic; c Institute of Physiology of the Czech Academy of Sciences, Videnska 1083, 142 20 Prague, Czech Republic; d Department of Biology, Faculty of Education, J. Selye University, Slovakia; e John Van Geest Centre for Brain Repair, University of Cambridge, Cambridge, United Kingdom; f School of Biomedical Sciences, Faculty of Biological Sciences, University of Leeds, United Kingdom; g Institute of Physics of the Czech Academy of Sciences, Prague, Czech Republic

**Keywords:** Perineuronal net, Hyaluronan, Memory, Neuroplasticity, Extracellular matrix

## Abstract

Hyaluronan (HA) is a core constituent of perineuronal nets (PNNs) that surround subpopulations of neurones. The PNNs control synaptic stabilization in both the developing and adult central nervous system, and disruption of PNNs has shown to reactivate neuroplasticity. We investigated the possibility of memory prolongation by attenuating PNN formation using 4-methylumbelliferone (4-MU), an inhibitor of HA synthesis. Adult C57BL/6 mice were fed with chow containing 5% (w/w) 4-MU for 6 months, at a dose ~6.7 mg/g/day. The oral administration of 4-MU reduced the glycosaminoglycan level in the brain to 72% and the spinal cord to 50% when compared to the controls. Spontaneous object recognition test (SOR) performed at 2, 3, 6 and 7 months showed a significant increase in SOR score in the 6-months treatment group 24 h after object presentation. The effect however did not persist in the washout group (1-month post treatment). Immunohistochemistry confirmed a reduction of PNNs, with shorter and less arborization of aggrecan staining around dendrites in hippocampus after 6 months of 4-MU treatment. Histopathological examination revealed mild atrophy in articular cartilage but it did not affect the motor performance as demonstrated in rotarod test. In conclusion, systemic oral administration of 4-MU for 6 months reduced PNN formation around neurons and enhanced memory retention in mice. However, the memory enhancement was not sustained despite the reduction of PNNs, possibly due to the lack of memory enhancement training during the washout period. Our results suggest that 4-MU treatment might offer a strategy for PNN modulation in memory enhancement.

## Highlights

• Removal of perineuronal nets (PNNs) reactivates neuroplasticity.
• Oral administration of 4-methylumbelliferone (4-MU) reduces PNNs.
• PNN reduction leads to enhancement in recognition memory in mice.
• The memory effect is not sustained likely due to a lack of memory training.

## Abbreviations

4-MU: 4-methylumbelliferone
CNS: central nervous system
CSPGs: chondroitin sulfate proteoglycans
CTCF: Corrected Total Cell Fluorescence
ECM: extracellular matrix
GAGs: glycosaminoglycans
HAS: hyaluronan synthase
Hapln: hyaluronan and proteoglycan link protein
HA: hyaluronan
PNNs: perineuronal nets
SA: Spontaneous alternation test
SOR: spontaneous object recognition test
WFA: *Wisteria floribunda* agglutinin

## 1 Introduction

The extracellular matrix (ECM) is a three-dimensional network that provides structural and biochemical support to surrounding cells. In the brain, ECM molecules exist both as a diffuse and a condensed forms, and play important roles in neuronal development, plasticity, and pathophysiology (Miyata and Kitagawa, 2017).

The most prominent form of condensed ECM in the central nervous system (CNS) is perineuronal nets (PNNs), which surround the soma and proximal dendrites of various neuronal subpopulations and have been shown to be responsible for the synaptic stabilization and receptor clustering, ultimately limits plasticity (Frischknecht et al., 2009, Pyka et al., 2011, Fawcett et al., 2019). PNNs have been shown to contribute to the multiple physiological brain functions, including learning and memory, and are involved in many disorders or pathologies, such as recovery from the spinal cord injury, schizophrenia, neurodegenerative diseases, epilepsy, autism and drug addiction (Sorg et al., 2016, Pantazopoulos and Berretta, 2016, Bozzelli et al., 2018).

Manipulation or disruption of PNNs have been shown to reactivate neuroplasticity, and improve learning and memory associated with the aging or neurological diseases, such as Alzheimer disease (Duncan et al., 2019). Several proof-of-concept approaches have been developed to manipulate or remove PNNs to increase the neuroplasticity, memory and CNS repair. These include enzymatic degradation of ECM molecules (Romberg et al., 2013, Kwok et al., 2008, Howell and Gottschall, 2012), antibodies blocking PNN inhibitory action (Yang et al., 2017), or genetic modifications such as knockout models with gene deletion of various PNN components (Romberg et al., 2013, Carulli et al., 2010). Genetic attenuation of PNNs through specific knock down of link protein *hapln1* in the CNS prolongs memory for familiar objects. Similarly, a localized digestion of PNNs with chondroitinase ABC, an enzyme that degrades the chondroitin sulfate proteoglycan components of ECM also enhances object recognition memory (Romberg et al., 2013).

In order to enhance PNN plasticity as a treatment, it is advantageous to develop a specific drug for this purpose. A promising strategy could be using small molecule compounds to prevent the biosynthesis of PNN components, such as hyaluronan (HA). In the CNS, HA is a major component of the loose ECM and PNNs, and has been shown to regulate the differentiation of neural stem cells (Su et al., 2017). It forms a backbone of mesh‐like structure which holds the PNNs on neuronal surface and allows for the binding of other important components such as link proteins (*haplns*), chondroitin sulfate proteoglycans (CSPGs) and tenascin (Fawcett et al., 2019, Carulli et al., 2010, Kwok et al., 2010). Prevention of HA biosynthesis might be the effective target for PNN manipulation.

A well-established small molecule inhibitor of HA synthesis is 4-methylumbelliferone (4-MU), a coumarin derivative (7–hydroxy‐4–methylcoumarin). 4-MU acts as a competitive substrate for uridine diphosphate (UDP)-glucuronyltransferase, an enzyme involved in HA synthesis, and causes depletion of cellular UDP-glucuronic acid, which is a building component of HA (Kakizaki et al., 2004). It was also shown that 4-MU downregulates the mRNA levels of HA synthase 2 and 3 (Kultti et al., 2009) or mRNA for UDP-glucose pyrophosphorylase and dehydrogenase (Vigetti et al., 2009). In addition, other mechanisms of 4-MU actions independent of inhibition of HA synthesis has also been suggested (Ishizuka et al., 2016).

4-MU, also called hymecromone, is already approved in multiple countries in Europe as choleretic and antispasmodic drug. Moreover, 4-MU prevents the up-regulation of HA and its beneficial effect have been reported in the treatment of cancers, and various animal models of inflammatory and autoimmune diseases, with the pathology associated with HA over-production (Nagy et al., 2015). Feeding of 4-MU was protective in experimental autoimmune encephalomyelitis model of multiple sclerosis in mice, where it modulates T-cell responses toward a FoxP3 + regulatory T-cell phenotype, related with disease prevention, and prevented suppression of the protective chemokine CXCL12 in CNS tissue (Kuipers et al., 2016, Mueller et al., 2014). In the model of lung staphylococcal enterotoxin B or LPS-induced lung inflammation in mice, 4-MU treatment led to a reduction in HA levels, and decreased lung permeability and pro-inflammatory cytokine production (McKallip et al., 2015, McKallip et al., 2013). 4-MU treatment also inhibited development of rheumatoid arthritis (Yoshioka et al., 2013) and prevented liver fibrosis (Andreichenko et al., 2019).

In this study, we investigated the effect of 4-MU on the PNNs, the structures where HA plays an indispensable role. We observed a reduction of HA after a 6-month oral treatment of 4-MU. In addition, there is a reduction of PNNs with a concomitant enhancement in plasticity and an improvement in recognition memory with the use of spontaneous object recognition test in adult C57BL/6 mice. An assessment of the PNN structures around hippocampal neurons showed a reduction in PNN length on neurites and on the number of arborization. Histopathological studies showed a mild atrophy in articular cartilage but it did not affect the motor function of the mice as demonstrated in rotarod test.

## 2 Methods

### 2.1 Animals

Three months old mice C57BL/6JOlaHsd strain (males n = 12; females n = 28) (Jackson Laboratory, Bar Harbor, Maine) were used in this study. All experiments were performed in accordance with the European Communities Council Directive of 2020 September 2010 (2010/63/EU), regarding the use of animals in research and were approved by the Ethics committee of the Institute of experimental medicine CAS, Prague, Czech Republic. Animals had unrestricted access to food and water and were maintained on a 12 h light/dark cycle (lights off at 7:30 p.m.). All behavioural testing was conducted during the light phase of the cycle.

In the first group of mice (n = 16, female), we tested the different flavors of chow with 5% (w/w) 4-MU at a dose ~6.5 mg/g/day. The animals were randomly divided into 4 groups (n = 4 per group). 4 mice with the same treatment will be group-housed in one cage to avoid social isolation. Mice was fed *ad libitum* with 5% (w/w) 4-MU in Western diet (1.25% Cholesterol, Sniff GmbH, Germany) with chocolate, orange and banana flavor (4-methylubilliferone, DbPharma France). The mice and the consumed chow were weighed every 3 days for 28 days and compared with a control non-flavored chow (Western diet, Sniff GmbH, TD.88137, Germany) without 4-MU. While each mice was weighed individually (i.e. four data per time point from 4 mice), chow was being weighed from the food hopper and averaged for the calculation of chow consumption (i.e. one data per time point).

Second group of animals (n = 24) was fed *ad libitum* with 5% (w/w) 4-MU (8 male and 8 female) or control chocolate flavored Western diet (4 male and 4 female) for 6 months. Part of the animals were sacrificed directly after 6 months of the 4-MU treatment (4-MU n = 8, control n = 4) and organs (brain, spinal cord, spleen, liver, kidney and cartilage tissue) were dissected and used for biochemical, histological or qPCR analyses.

The rest of the animals were fed with 4-MU (n = 8) or a control chow (n = 4) for the 6 months and then with the control chow for the next 4-weeks to evaluate the wash-out effect. The animals were tested behaviorally for the memory and motor functions and sacrificed after 7 months when organs (brain, spinal cord, spleen, liver and cartilage tissue) were dissected and used for histological, immunohistochemical and qPCR analyses.

### 2.2 Behavioral tests

To observe the effect of 4-MU, series of memory and locomotor functional tests were performed. Spontaneous recognition tasks were used for the evaluation of changes in memory and plasticity of the brain. Rotarod and grip test were used for verifying the impact of the drug on the motor functions, mobility of joints and muscular degeneration.

#### 2.2.1 Spontaneous alternation test (SA)

SA tests hippocampus-dependent behavior as an attention toward novelty and spatial memory. Test was performed in a Y-maze with arms of identical dimensions and spaced at 120° angle from each other as described previously for rats (Ennaceur and Delacour, 1988, Winters et al., 2004) and mice (Bartko et al., 2011). The apparatus had high homogenous white non-transparent walls constructed from extruded polystyrene. All walls were 30 cm high, and each arm was 15.5 cm in length and 8 cm wide. A lamp illuminated the apparatus, and a video camera were mounted 40 cm above the apparatus to record trials. Each mouse was placed at the center of the Y-maze and allowed to move freely to explore the empty apparatus for 5 min. Over the course of multiple arm entries, the animals typically show a tendency to enter a less recently visited arm. The numbers of arm entries (activity) and alternation were recorded. Alternation was defined as an entry which was different from the two previously entered arms; no alternation was defined if the mouse went back to either of the two arms just previously visited. Percentage of alternation was calculated using this formula:

%Alternation=(Numberofalternation/Totalnumberofentries)×100%

#### 2.2.2 Spontaneous object recognition task (SOR)

**Fig. 1:**
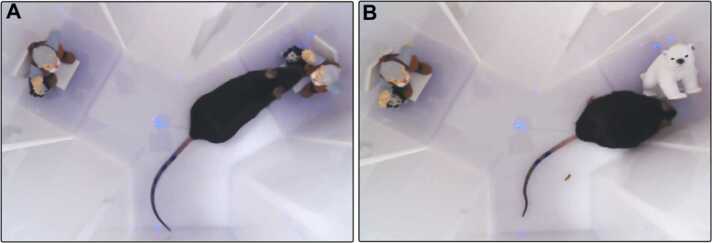
The sample (a) and choice (b) phases of the SOR test used for the memory retention analysis.

In the SOR test, two familiar and one novel objects in the Y maze were used to determine the recognition memory of the mice. One arm of the Y maze was used as a start arm and the other two arms were used to display the objects (randomly shaped junk objects, dimensions ~10 cm × 5 cm × 5 cm). All mice were habituated to the Y maze in two-day sessions. To explore the Y-maze with the dimensions used for SOR test where 2 arms were 10 cm in length, 8 cm wide and the start arm was 4.5 cm in length and 8 cm wide. The following test sessions were separated by a minimum of 48 h. Each test session consisted of a sample phase and a choice phase. In the sample phase, two identical objects (Fig. 1A) were placed at the end of each arm. The animal was left to explore the objects for 5 min. The choice phase followed after a delay of 3 or 24 h which the animal spent in the home cage. The choice phase was procedurally identical to the sample phase, except that the object in one of the arms was replaced by a novel object whereas the other arm contained the familiar object (Fig. 1B). A different object pair was used for each session for a given animal. Time spent with object pairs were counter-balanced 1 h after exposition and objects with no preferences were selected for the experiment. The performance was recorded, and the time spent in exploring objects from each animal was assessed from the video recordings of the sample and choice phases. Occasions where an animal climbed or sat on an object were discarded. For the choice phase, a discrimination score was calculated by dividing the difference in exploration of the novel and familiar objects by the total object exploration time. Therefore, a score of 1 corresponded to exploration of the novel object only, whereas a score of 0 corresponded to the mouse equally exploring the novel and the familiar object (Bralett’s test, one-way ANOVA).

#### 2.2.3 Rotarod and grip test

Rotarod and grip test were performed after 6 months of 4-MU treatment. A rotarod machine with automatic timers and falling sensors with a 7 cm diameter drum (ROTA-ROD 47700, UGO BASILE S.R.L., Italy) was used for this test. Before the training sessions, mice (control n = 4; 4-MU n = 8) were habituated to stay on the stationary drum for 60 s and pre-trained for 5 rotations per minute (RPM) for 120 s. In the test phase, animals were tested for 300 s with 10 RPM in 3 different days follow the training.

For the grip test, grip strength test (BIO-GS3, Bioseb, Vitrolles, France) was used. Mice (control n = 4; treated n = 8) were test for the grip of hind limbs in 3 sessions in the 3 subsequent days.

### 2.3 Tissue processing and histology

The animals used for immunohistochemical and histological analysis (n = 4 for control, n = 7 for treated group) were deeply anesthetized with an intraperitoneal injection of overdose chloral hydrate (Sigma-Aldrich, St. Louis, Missouri, USA) and intracardially perfused with phosphate buffered saline (PBS), followed by 4% paraformaldehyde in 0.1 M phosphate buffer. Head was post-fixed in paraformaldehyde for 1 week. After the brain was dissected from the skull, it was transferred to solutions with increasing concentration of sucrose from 10% to 30% (w/v). Brain was then embedded with O.C.T compound (00411243; VWR), frozen and a series of 20 µm thick coronal sections were cut on cryostat (CryoStar NX70 ThermoScientific, Massachusetts, USA), placed on the glass tissue slides and stored at − 20 °C.

Before staining, brain coronal sections were permeabilized with 0.5% (v/v) Triton X-100 in PBS for 20 min at room temperature. Lipofuscin autofluorescence was quenched by ammonium acetate buffer 50 mM with CuSO_4_. To block of endogenous biotin signal we used endogenous biotin blocking kit (#Ab64212, Abcam, Cambridge, GB).

PNNs were visualized by the *Wisteria floribunda* agglutinin (WFA), which specifically labels *N*-acetylgalactosamine beta 1 residues of glycoproteins within the ECM of the neurons (1:150; biotinylated WFA, L1516, Lectin from Wisteria floribunda, Sigma Aldrich), and aggrecan (1:150; Anti-aggrecan, rabbit polyclonal, #AB1031, Merck), one of the CSPGs bound to the HA chains in PNNs. Blocking of non-specific binding of aggrecan antibody and WFA was performed using 10% chemiblocker (#2170, Merck, Darmstadt, Germany) in 1X PBS with Triton 0.2% (v/v) at room temperature for 2 h.

Primary and secondary antibodies were diluted in the solution of the same composition as an immuno-blocking solution. Primary antibodies against aggrecan and biotinylated WFA were incubated overnight at 4 °C. WFA was visualized using streptavidin conjugated with Alexa Fluor 488 (1:200; S32354, Life Technologies, USA). Aggrecan was stained by goat-anti-rabbit secondary antibody conjugated with Alexa Fluor 594 (1:300; A11012, Life Technologies, USA). Nuclei were stained using DAPI (1:1000; D1306, Invitrogen). Slices were coverslipped mounted with the anti-fading medium Vectashield (#H-1000; Vector Laboratories, Burlingame, USA).

### 2.4 PNN analysis by immunohistochemistry

Images of fluorescent WFA staining was taken by Leica microscope (Leica DMI 6000B, ‎Wetzlar, Germany) using a 20× objective and analysed by ImageJ™ software (NIH). The number of WFA positive (WFA+) neurons was determined in the hippocampal area from four brain sections from each animal (n = 4 per group). The intensity of WFA staining was measured from WFA+ neurons in the CA1–3 area of the hippocampus. The value of the intensity was calculated as: Corrected Total Cell Fluorescence (CTCF) = Integrated density of selected cell – integrated density of background readings. In addition, the total number of cells surrounded by PNNs was counted from the number of WFA+ neurons in the CA1-CA3 area. For statistical analysis Bartlett’s test, one-way ANOVA were used.

Fluorescent images of neurons (40 neurons per animal, n = 4 per group) stained for aggrecan were taken from CA2/CA3 area using Confocal Microscope Zeiss LSM 880 Airyscan (Zeiss, Oberkochen, Germany) with the 20x objective. Tracing of aggrecan stained PNNs was performed using fluorescent microscope (Leica DMRXA microscope) with Neurolucida2019 software (MBF Bioscience, Williston, Vermont, United States). The 3D tracing was performed manually, starting with neuronal soma. PNNs on dendritic branches were traced with a simultaneous slow movement along the z-axis to keep the traced segment at maximum focus. The final PNN tracings of particular neurons were saved and Neurolucida Explorer 2019 (MBF Bioscience) was used to conduct a detailed morphological structure analysis. The obtained data were further exported and statistically processed with GraphPad Prism Software (8.0). Differences between groups were analysed using Kruskal Wallis test, followed by Dunn’s multiple comparisons test.

### 2.5 Glycosaminoglycan (GAG) extraction and quantification

GAG analyses were performed on 3 animals per group. GAGs purification was performed according to the protocol from (Lin et al., 2011). Briefly, brains and spinal cords were dissected, frozen on dry ice and stored at − 80 °C until purification. Acetone powder of the sample was prepared by homogenising the samples with chilled acetone and drying by desiccation at 4 °C. The dried powder was re-suspended in pronase solution (in buffer containing 0.1 M Tris-acetate, 10 mM calcium acetate, pH 7.8) at 37 °C overnight. Samples were then centrifuged, digested protein in the supernatant was precipitated with trichloroacetic acid (5% v/v final concentration). The supernatant was collected after centrifugation and was washed with diethyl ether. The GAGs present in the aqueous phase were precipitated with sodium acetate (5% w/v final concentration) and ice-cold ethanol (75% v/v final concentration) at 4 °C overnight. Precipitated GAG was recovered by centrifugation and dried at 4 °C. This whole GAG preparation was redissolved in water and stored at − 20 °C. The total GAG content in each sample were quantified using cetylpyridinium chloride (CPC) turbidimetry assay (Manley and Hawksworth, 1966). Chondroitin sulfate-A (Sigma Aldrich) was used to set up the standard curves.

### 2.6 Gene expression analysis

Changes in the mRNA expression of genes related to the HA synthesis (*has1, has2, has3*), hyaluronidases (*hyal1, hyal2, hyal3, tmem2, spam1*), chondroitin sulfate proteoglycans (*acan, bcan, ncan, vcan*), tenascine-C (*tnc*), synaptic growth (*ngf, gria2, syp*) and receptors for HA (*CD44, lyve1*) were determined after 6 months of the treatment for 4-MU and 4 weeks after the treatment by quantitative real‐time PCR (qPCR), and plotted against the control untreated group of animals. RNA was isolated from paraformaldehyde‐fixed frozen tissue sections using the High Pure RNA Paraffin Kit (Roche, Germany). RNA amounts were quantified using NanoPhotometer P 330 (Implen, Germany). Isolated RNA was reverse transcribed into complementary cDNA using the Transcriptor Universal cDNA Master (Roche) and T100 Thermal Cycler (Bio‐Rad, USA). The qPCR reactions were performed using cDNA solution, FastStart Universal Probe Master (Roche) and TagMan Gene Expression Assays (Life Technologies, Carlsbad, CA, USA) (Supplementary Table 1).

The qPCR was carried out in a final volume of 10 µL containing 45 ng of extracted RNA. Amplification was performed on the real‐time PCR cycler (QuantStudio 6, ThermoFisher, Massachusetts, USA). All amplifications were run under the same cycling conditions: 2 min at 50 °C, 10 min at 95 °C, followed by 40 cycles of 15 s at 95 °C and 1 min at 60 °C. All amplifications were run in duplicates and a negative control (water) was included in each array; with Gapdh as a reference gene. A log2 scale was used to display the symmetric magnitude for up and down regulated genes. The values of non-treated animals were set as zero line. Differences between the treated and non-treated groups were analysed for statistical significance with ΔCt values level, using a one-way ANOVA test with Kolmogorov-Smirnof.

### 2.7 Histopathological evaluation

For histopathological evaluation, spleen, liver, and kidney were sampled and fixed for a minimum of 24 h in 4% paraformaldehyde in 0.1 M PBS. Samples were processed according to standardized protocol using autotechnicon Leica ASP 6P25 (Leica Biosystems Nussloch GmbH, Germany) and paraffin blocks using embedding station Leica EG 1150 H (Leica Biosystems Nussloch GmbH, Germany). Approximately 10 µm slices were cut on a rotary microtome Leica RM2255 (Leica Biosystems Nussloch GmbH, Germany), placed onto standard slides (Bammed s.r.o., Czech Republic) and stained with haematoxylin-eosin (DiaPath, Italy).

Articular cartilage samples were fixed for a minimum of 24 h in 4% paraformaldehyde in 0.1 M PBS. Samples were afterward washed 4 times with distilled water during 24 h. Cartilages were treated with water solution of 15% EDTA (w/v), pH 8.0, which was changed every 3 days for 2 weeks. The samples were rinsed with distilled water and treated with increasing concentration of sucrose (10–20–30% w/v) in PBS and sections were cut on cryostat (CryoStar NX70 ThermoScientific), placed on the glass tissue slides and stored at − 20 °C. The H&E stained tissue slices were observed and imaged using Zeiss microscope (Zeiss Axioskop 2 Plus, Zeiss, Germany).

### 2.8 Statistical analysis

Data are presented as mean ± standard error of mean (SEM), statistical significance was analysed using SigmaPlot V13 (Systat Software Inc., USA) and GraphPad Prism Software (8.0) with the *p ≤ 0.05, **p ≤ 0.01, ***p ≤ 0.001.

## 3 Results and discussion

### 3.1 Formulation for non-invasive oral administration of the 4-MU

PNNs present a promising treatment target for numerous brain diseases or CNS injuries. In this regard, 4-MU, a drug that inhibits HA synthesis, might have a wide potential in functional modulation of structures rich on HA. 4-MU is an approved drug for human consumption. Daily oral doses of 1200 mg/day for 3 months is well tolerated in humans without serious adverse effects and it serves as a dose for exploring new indications (Nagy et al., 2015, Trabucchi et al., 1986). However, to achieve and maintain an effective drug concentration in the organs such as the brain, a higher dose of 4-MU is required due to its rapid clearance and therefore low systemic bioavailability (< 3%) (Nagy et al., 2015). In this study, we used oral administration in a dose ~ 6.7 mg/g/day (5% w/w 4-MU in chow), which has been shown previously as therapeutically effective with a well-demonstrated tolerance (Nagy et al., 2015, Kuipers et al., 2016, Kuipers et al., 2016).

To avoid force feeding and facilitate spontaneous feeding behaviour, 4-MU (5% w/w) was mixed with different flavors (chocolate, orange and banana) in the chow and consumption was allowed *ad libitum*. To prevent the effect of weight loss in the animals after 4-MU consumption described before (Kuipers et al., 2016), we combined 4-MU treatment with the high fat diet recommended for rodents.

**Fig. 2:**
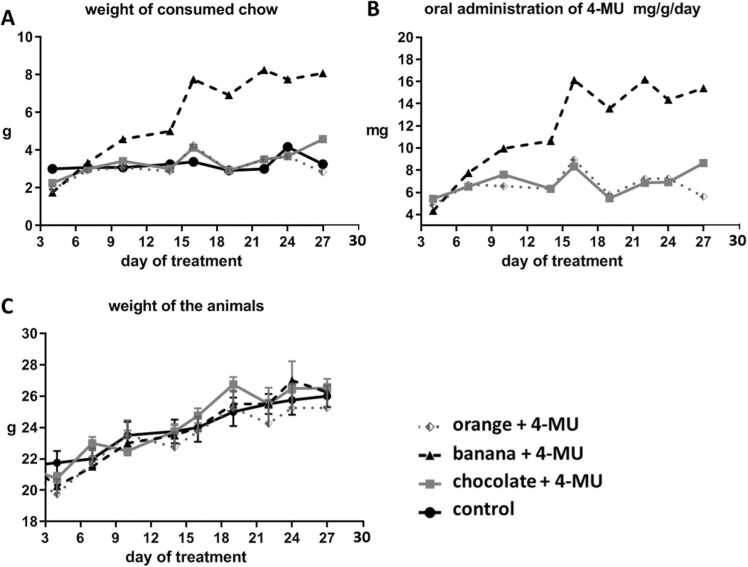
The effect of flavor on the amount of consumed chow and weight of animals. Mice were fed with the control or 5% (w/w) 4-MU chow with a chocolate, orange or banana flavor ad libitum for the 28 days. (A) Remarkable enhancement of consumed chow was observed for banana flavor compared with all other groups. (One Way ANOVA; Dunn's Method; p*** = 0.0001). (B) The corresponding amount of consumed 4-MU. (C) The weight of the animals did not differ in all groups. n = 4 per group. (One Way ANOVA; Dunn's Method; p*** = 0.0003).

We first compared the amount of consumed chow with different flavors and the weight of the animals for 28 days (Fig. 2). When we used the chocolate and orange flavored 4-MU chow, the weight of the consumed chow as well as the weight of the animals did not differ from the control non-flavored chow. Surprisingly, a remarkable increase in 4-MU chow consumption was observed for banana flavor compared to all other groups (p = 0.0001). On the other hand, the enhanced feeding preference of banana-flavored chow had no effect on the weight of the animals (Fig. 2C).

We showed that incorporating 4-MU in a chocolate or orange favored chow is an easy and non-invasive method to administer the drug to the animals. To make our study comparable to the previous work (Kuipers et al., 2016, Tsuchiya et al., 2020), we chose chocolate flavor chow for the following 6-months 4-MU treatment.

### 3.2 4-MU down-regulated GAGs in the CNS

**Fig. 3:**
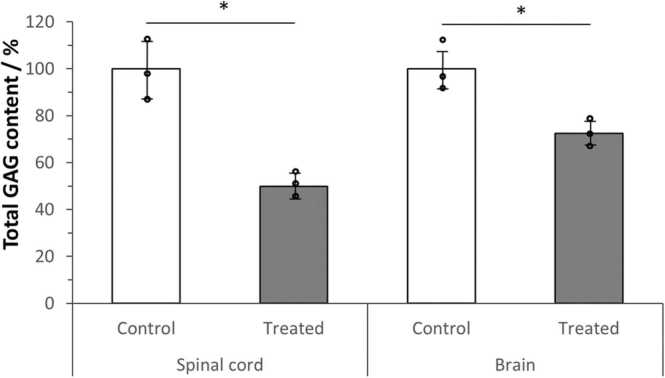
4-MU treatment has led to a reduction of GAGs in the CNS. Total GAG content (ug/mg of wet weight) was normalized to the quantity in the control treated mice. 4-MU treatment has led to a significant reduction of total GAG both in the spinal cords (from 100% to 49.93% ± 3.99%, p = 0.0127) and the brains (from 100% to 72.46% ± 4.51%, p = 0.0300). There is a stronger reduction in the spinal cord than in the brain (50.07% Vs 27.54%). n = 3, Student t-test, two tailed paired.

To determine the effect of systemic 4-MU treatment on the HA synthesis, we first investigated the amount of GAG reduction in the CNS. HA belongs to the family of GAGs, which also include heparin/heparan sulfate, chondroitin sulfate, dermatan sulfate, and keratan sulfate. GAGs were isolated and analysed following our previously published protocol (Lin et al., 2011). In comparison to control fed mice, 4-MU treatment has led to a significant reduction of total GAGs by 50% in the spinal cord and 28% in the brain (Fig. 3). The stronger reduction of GAGs in the spinal cord could be due to differences in HA metabolism between the brain and spinal cord. The results, however, confirm that oral treatment of 4MU could effectively down-regulate HA in the CNS.

### 3.3 4-MU improved memory retention

To investigate the effect of 4-MU in neuroplasticity, we used a set of behavioral tests focused on the short-term memory. Behavioural tests were started at 2 months after 4-MU treatment. This is mainly due to our previous observation in spinal cord injury that behavioural benefits from 4-MU treatment only appear at 6–7 weeks after treatment (Kwok et al., 2021). We thus decided to start our behavioural testing at 2 months after 4-MU treatment.

**Fig. 4:**
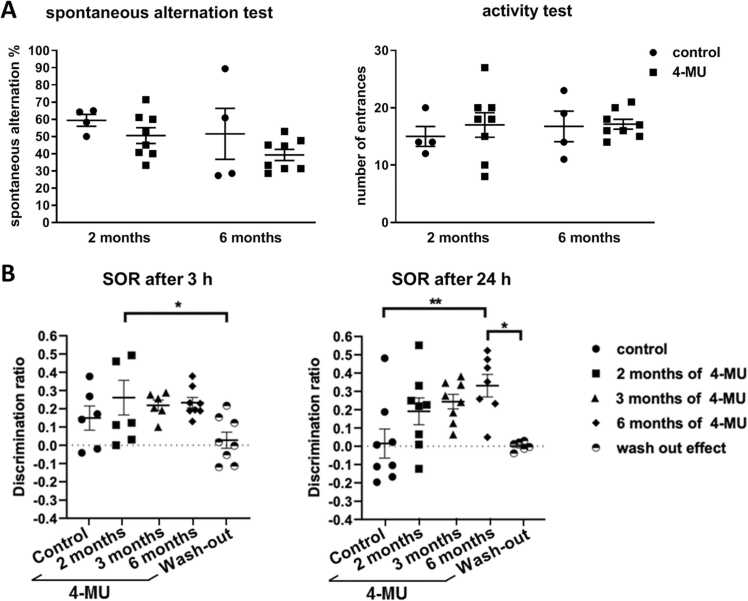
The spontaneous alternation (SA) and activity test after 2 and 6 months of the 4-MU treatment. (A) SA as well as an activity test did not show any significantly changes after the administration of the 4-MU treatment compared to the control animals. (B) SOR test was done after 2/3/6 month of the 4-MU treatment and then after 1 month (washout). A significant enhancement of SOR score was found in the 3 h delay in animals treated with 4-MU after 2 months compared to the wash-out group and in 24 h delay after 6 months of treatment compared to control and wash out group of animals. SOR score decreased to the control level after 1 month wash-out, which suggests that the effect of 4-MU is temporal. *p < 0.05; **p < 0.01, One Way ANOVA; Tukey’s multiple comparison test.

First, we examined the spontaneous alternation (SA) after 2 and 6 months of 4-MU treatment (Fig. 4A). This test is based on counting the alternation in three consecutive entrances to the arms which were not visit before, and it quantifies the willingness of the rodents to explore new environment as well as short term spatial memory. There is no difference in the total number of entries or alternations between the groups. This suggests that 4-MU did not affect the exploratory behaviour of the mice.

We then tested recognition memory after 2, 3 and 6 months of 4-MU treatment and after 1 month of 4-MU wash-out in the SOR test. This test is based on the tendency of rodents to interact more with a novel object than with a familiar object and animals are tested at various times after exposure to the objects to measure memory persistence. While 4-MU treatment showed a trend of memory enhancement in all time points of the treatment, significantly enhancement in 3-hour memory retention was found after 2 months of treatment compared to the washout group, and in 24-hour memory retention after 6 months of the treatment as compared to the control and washout group (Fig. 4B). Similar observation of memory enhancement in 24-hour object recognition tests have been previously observed with knock-out mice lacking the *Hapln1* gene, which encodes a link protein essential for PNN formation in the CNS, as well as after enzymatic degradation of PNN structures with chondroitinase ABC in the perirhinal cortex (Romberg et al., 2013). Our results suggest that oral treatment of 4-MU could improve memory retention via PNN down-regulation.

To investigate whether the effect of 4-MU is permanent, we performed SOR test one month after the termination of the 6-month 4-MU treatment (washout effect). We found that the memory-enhancing effect of 4-MU did not persist 1 month after the end of the 4-MU treatment, and the SOR score returned to the values as before the treatment. While the high level of neuroplasticity induced by the reduction of PNNs after 4-MU treatment facilitates memory retention, the lack of persistent memory benefits in the wash-out group suggests that a constant low PNN level is required. Alternatively, the observation in the wash-out group could be due to the lack of memory training during the wash-out period (Fig. 4). In addition, the results also suggest the involvement of other PNN-independent mechanism in the control of object recognition memory. Indeed, HA has been shown to differentially regulate the renewal, proliferation and differentiation of neural stem cells in the hippocampus (Su et al., 2017). It could be that the persistence low level of HA induced by the prolonged 4-MU administration hampers hippocampal neurogenesis which is instrumental in memory acquisition and formation.

### 3.4 4-MU reduced PNNs in the hippocampal area

It has been shown previously that recognition memory is related to the hippocampus and adjacent cortical areas including entorhinal, perirhinal, and parahippocampal cortex that are involved in normal memory function (Baxter, 2010). In the previous reports, reduction of PNNs by chondroitinase ABC in the perirhinal cortex altered SOR task (Romberg et al., 2013, Yang et al., 2015). These structures are highly integrated; the perirhinal cortex is involved in object recognition after short retention intervals, while the hippocampus is mainly associated with spatial navigation and in the encoding, consolidation and retrieval of non-spatial memory including object recognition memory. We thus investigated the effect of 4-MU in PNN expression in the hippocampus.

**Fig. 5:**
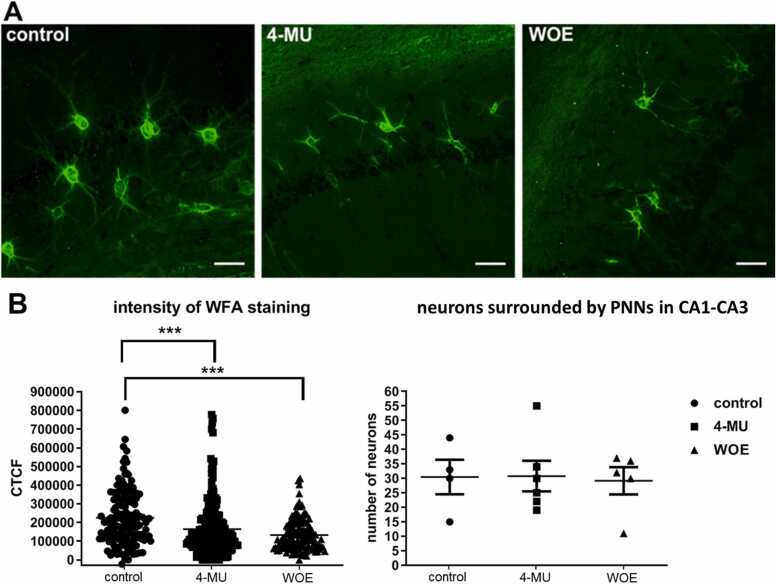
(A) Representative images of immunofluorescence staining of WFA in CA1-CA3 of the hippocampal area in the control, 4-MU treated for 6 months and 4-MU treated animals after 1 month of the wash-out. (B) Intensity of WFA staining and total number of WFA positive cells determined in the hippocampal area (CA1–CA3). A significant decline of the WFA intensity staining was found after 6 months of 4-MU treatment and it persisted after 1 month of the wash-out when compared with the control. n = 4 per group, ***p < 0.001; one-way ANOVA test. WOE – wash-out effect. Scale: 50 µm.

Here, we confirmed a reduction of PNNs after 4-MU treatment in the hippocampus. We used WFA staining that selectively labels the N-acetylgalactosamine-beta-1 residues of glycosaminoglycans of PNNs in the ECM to first evaluate the effect of 4-MU in the intensity of PNN and the total number of the neurons surrounded by PNNs (Fig. 5). While the total number of WFA positive neurons remains similar, there is a significant decrease in the WFA intensity around neuronal bodies in both the 4-MU treated and the wash-out groups (Fig. 5B).

**Fig. 6:**
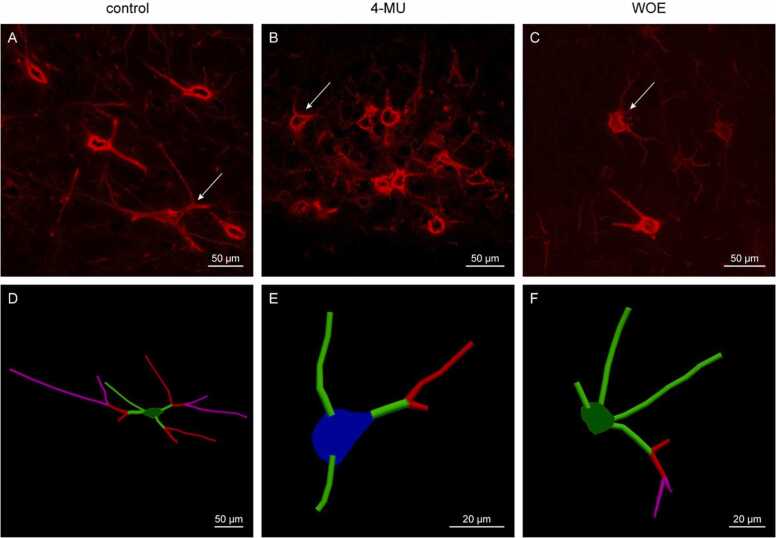
(A–C) Illustrations of aggrecan stained PNNs in neurons in the hippocampal CA2/CA3 area in the (A) control, (B) 4-MU after 6 months of treatment and (C) WOE group. (D–F) Examples of PNN tracing in the (D) control, (E) 4-MU and (F) WOE. Arrows in A, B, C depict PNNs of neurons traced in D, E, F. WOE-washout effect. Scale: (A–D) 50 µm, (E, F): 20 µm.

**Fig. 7:**
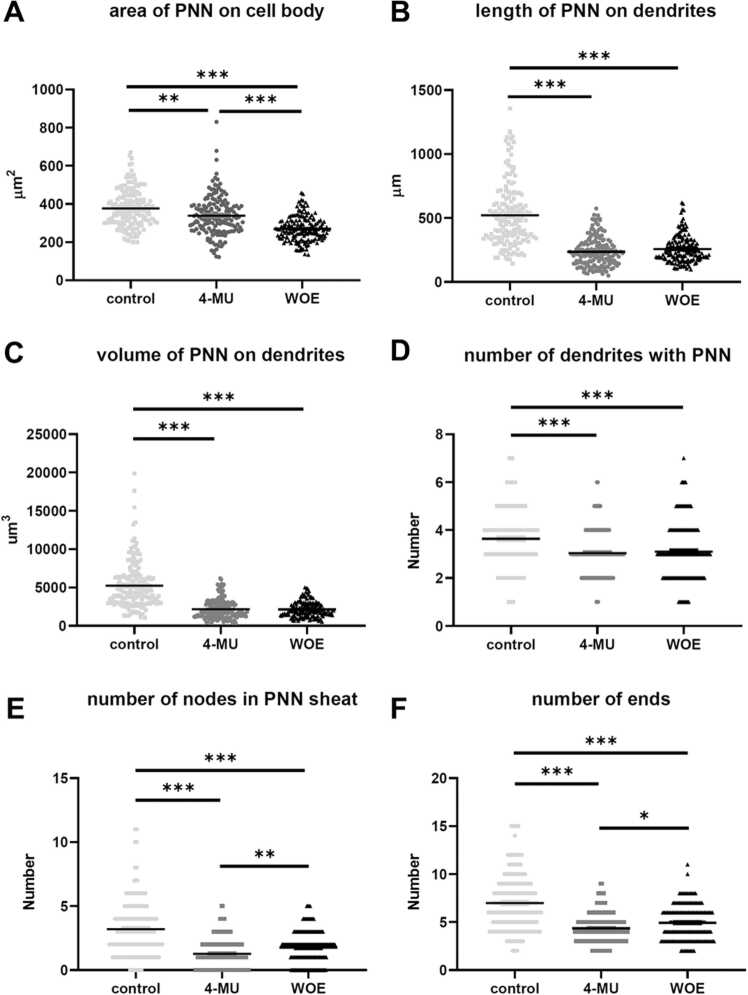
Morphological parameters of PNNs in the hippocampal CA2/CA3 area in the control, 4-MU after 6 months of treatment, and WOE group. n = 4 per group, 40 neurons per animal, ***p < 0.001, ** p < 0.01, *p < 0.05, Kruskal Wallis test, followed by Dunn's multiple comparisons test.

Aggrecan is a CSPG which is present in almost all PNN-positive neurons in the brain and it forms an integral structure of the PNNs by binding to HA chains through the Haplns (Kwok et al., 2011, Galtrey et al., 2008). Previous study has shown changes in dendritic length and arborization during plasticity. To investigate the effect of 4-MU in dendritic plasticity, we performed immunohistochemical staining for aggrecan and quantified the dendritic arborization of PNNs around individual neurons in the hippocampus (Figs. 6 and 7). The single cell analysis using the aggrecan staining of hippocampal neurons after 6 months of 4-MU treatment revealed a reduction in the area and volume of PNNs per neuron, and the length of PNN on dendrite (Fig. 7, A–C). Analysis of the arborization also found a reduction in dendritic complexity covered by the PNNs, this is reflected in the number of arborization, nodes and ends of the dendrites (Fig. 7, D–F). Similar to WFA staining, the aggrecan staining around neurons did not recover after 1 month of 4-MU washout (Fig. 7).

These results confirmed that HA is an essential component for PNN formation and that an inhibition of HA synthesis leads to a significant reduction in PNN morphology in hippocampal neurons. However, this study focused on the hippocampal region of CA2/CA3 with the most pronounced aggrecan staining, and therefore the ubiquitous effect of 4-MU on total PNN in other areas have to be confirmed in a future study.

The results suggest a correlation of recognition memory enhancement and PNNs reduction after 6 months of 4-MU treatment. This finding also agrees with previous report which demonstrated that improvement of recognition memory is related to attenuated PNNs in the hippocampus and its associated area such as perirhinal cortex (Romberg et al., 2013). Removal of hippocampal PNNs has been previously shown to disrupt contextual and trace fear memory, and a disruption of PNNs in the medial prefrontal cortex impaired long-term trace and conditioned stimulus-elicited fear memory in the trace fear conditioning task (Hylin et al., 2013). Notably, loss of PNNs in several brain regions has been suggested to contribute to cognitive impairment in disorders such as schizophrenia (Pantazopoulos and Berretta, 2016, Howell and Gottschall, 2012, Lewis et al., 2012) and PNN abnormalities are also associated with other neurological diseases (Wen et al., 2018). The opposing data demonstrate the complexity of PNNs in memory regulation.

It should be noted that much of the brain ECM exists as the diffuse compartment, and condensed ECM including PNNs represents a small proportion of the total ECM. Therefore, systemic inhibition of HA by 4-MU, which lacks specificity, can also cause the alteration of diffuse ECM. In this study, systemic HA inhibition caused a reduction of PNNs, which correlated with enhanced memory retention. On the other hand, one month after the end of 4MU treatment, the attenuation of PNNs still persisted, while the memory enhancing effect disappeared. This suggests that other PNN-independent, but ECM-related, mechanism is involved in the control of object recognition memory. HA and its receptor CD44 have been previously shown to regulate the hippocampal neurogenesis and hippocampal-dependent spatial memory (Su et al., 2017, Raber et al., 2014). It could be that the persistence low level of HA induced by the prolonged 4-MU administration hampers hippocampal neurogenesis which is instrumental in memory acquisition and formation.

Nonetheless, these data suggest that other factors in addition to PNNs might be involved in plasticity recovery after the treatment with 4-MU.

### 3.5 Changes in gene expressions using RT-qPCR

**Fig. 8:**
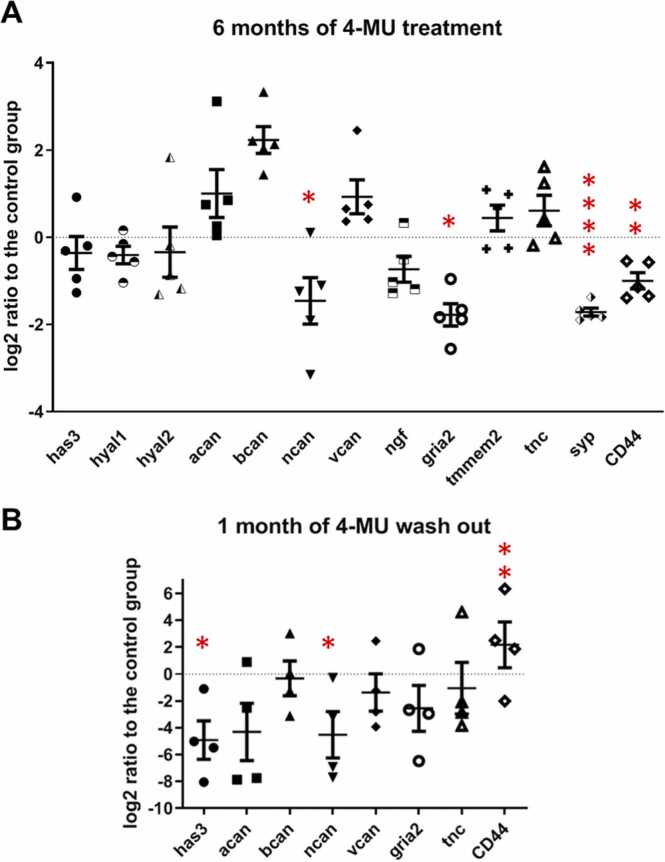
mRNA expression of selected genes after(A) 6 months of 4-MU treatment and (B) 1 month of 4-MU wash-out. Significant changes of expression were observed in neurocan (ncan), glutamate ionotropic receptor AMPA type subunit 2 (gria2), synaptophysin (syp), (CD44) with 6 months of treatment, and in hyaluornan synthase 3 (has3), ncan and CD44 in the WOE group when compared with the control group. n(control) = 4, n(4-MU) = 5, n(WOE) = 4. * p ≤ 0.05; ** p ≤ 0.01; *** p ≤ 0.001; ****p ≤ 0.0001 vs. control, one-way ANOVA; Student-Newman-Keuls Method.

To examine the effect of 4-MU treatment on the expression of genes related to HA metabolism (*has1–3*, hyal1–3, *tmmem2*) and signalling (*CD44*) in the brain and neuroplasticity (*ngf, gria2, syp*), we used RT-qPCR analysis (Fig. 8). HA is synthesized by transmembrane enzymes HAS1–3 and extruded from the cell surface where it forms a PNN when interact with other soluble PNN molecules (Kwok et al., 2010). Each HAS isoform shows different enzyme activities and variable requirements for the cellular supply of UDP-sugars in vivo. HAS1 seems to produce low level of hyaluronan in cells with a low content of the nucleotide sugars, HAS3 produces HA at a high speed even with minimum substrate content (Rilla et al., 2013). In the cerebellum, HAS2 and HAS3 are produced by cerebellar PNN-bearing neurons (Carulli et al., 2006). On the other hand, it has been demonstrated that the genetic ablation of HA synthesis in the brain of *Has*3^−/−^ mice revealed no detectable changes in the structure or composition of PNNs (Arranz et al., 2014), this could be due to compensatory regulation of other HASs. *In vitro*, 4-MU treatment downregulated HA biosynthesis and expression of HAS2 and/or 3 in various cancer cell lines (Kultti et al., 2009, Saito et al., 2013), bone marrow (Goncharova et al., 2012), or human articular chondrocytes (Ishizuka et al., 2016). We observed a reduced trend of *has3* expression in the 6 months treated group and a significant reduction in the WOE group. The expression of the other isoforms *has1* and *has2,* and the HA degrading enzyme *hyal3* was not detected (Fig. 8). Moreover, we also observed a significant reduction in the expression of *CD44*, the receptor of HA. These data agrees with previous report that 4-MU regulates *has* expression (Kultti et al., 2009) and HA metabolism leading to the reduced formation of PNNs.

In terms of other key PNN components (*acan, bcan, ncan, vcan, tnc*), there is a significant reduction in the mRNA expression of neurocan (*ncan*) after 6 months of 4-MU application as well as 1 month after wash-out. Neurocan is one of the key HA binding CSPGs in the PNNs, and is a potent inhibitor of neuronal and glial adhesion, and neurite outgrowth. A reduction in ncan could potential lead to a more permissive environment favouring neuroplasticity.

In order to determine the effect of 4-MU and its associated down-regulation of PNNs and ECMs in synaptic plasticity, we have also determine the expression for nerve growth factor (*ngf)*, AMPA-selective glutamate receptor 2 (*gria2*) and synaptophysin (*syp)*. We observed a significant down-regulation of *syp* after 6 months of 4-MU treatment. For *gria2*, the down-regulation was observed in both the 4-MU treated group and the wash-out group. This suggests that the effect of 4-MU on brain ECM was long-lasting and there is no recovery of PNN structures, neither on gene expression level or at the protein level even after 1 month after the end of treatment.

### 3.6 Histopathological examination after long-term 4-MU treatment

Apart from its weight bearing and lubricating function in cartilage, HA serves multiple physiological functions in the body. Systemic inhibition of hyaluronan synthesis by 4-MU (10 mg/g body wt) interfered with the protective function of the endothelial glycocalyx, facilitates leukocyte adhesion, subsequent inflammation, and progression of atherosclerosis in apolipoprotein E–deficient mice (Nagy et al., 2010). Disruption of HA production by intravenous injection of 4-MU (100 µL of 3 mM) decreased hematopoietic activity in vitro and lowers the migration of transplanted hematopoietic stem/progenitor cells into the marrow of irradiated mice (Goncharova et al., 2012). On the other hand, 4-MU diminished pro-inflammatory activation of articular chondrocytes and cartilage explants in vitro through a mechanism independent of HA (Ishizuka et al., 2016) or alleviates inflammatory responses in a model of murine arthritis (3 mg/g/day) and in human rheumatoid synovial fibroblasts (Yoshioka et al., 2013). Despite of the several studies of 4-MU administration on a variety of experimental disease models, a systematic histopathological study addressing the potential side effects of high doses of 4-MU is still lacking.

In the current study, we induced long-term non-specific inhibition of HA synthesis by the systemic 4-MU treatment in a dose ~ 6.7 mg/g. The dose is based on a previous publication (Nagy et al., 2015) but is much higher than the approved dose of hymecromone to treat biliary spasm. Notably, the LD_50_ of 4-MU for oral administration has been reported 2850 mg/kg in mice and 6200 mg/kg in rats (Bethesda (MD), 2021).

Despite exceeding the reported oral LD_50 (mice)_ by more than twice, no animal died during the 6 months of feeding in our study and there were no obvious behavioural sequelae. This suggests a much higher tolerability of oral administration of 4-MU than previously reported in the Pubchem database. The current LD_50_ for mice is reported from 1968 (Lontane et al., 1968), and drug preparation and impurity could have led to the observed difference. In our study, we used pharmaceutical grade 4-MU (5% w/w) that was showed tolerable in several previous studies, e.g., in a model of multiple sclerosis (Mueller et al., 2014), osteoarthritis (Tsuchiya et al., 2020) and diabetes (Sunkari et al., 2015). Undoubtedly, based on our data and recent reports, the LD50 for oral dose of 4-MU in mice needs revisiting.

To determine the potential adverse effect of systemic 4-MU administration on various tissues and their functions, we have performed histopathological observation of the cartilage, spleen, liver and kidney (Table S2), and investigated whether 4-MU treatment could impair the cartilage dependent motor capabilities, using rotarod and grip test after 6 months of the treatment.

**Fig. 9:**
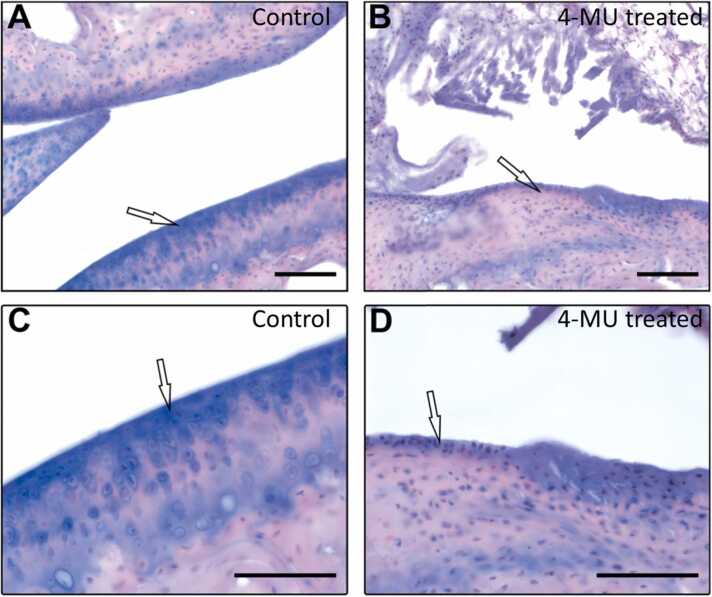
Alcian blue staining of the articular cartilage. (A, C) Articulation in normal bones is covered by standard thick hyaline cartilages. (B, D) Atrophy of the hyaline cartilage with thin bones, and trabecula with residual, intermittent regenerating hyaline cartilages are observed after 4-MU treatment. Scale bar: A,B: 100 µm, C,D: 10 µm.

**Fig. 10:**
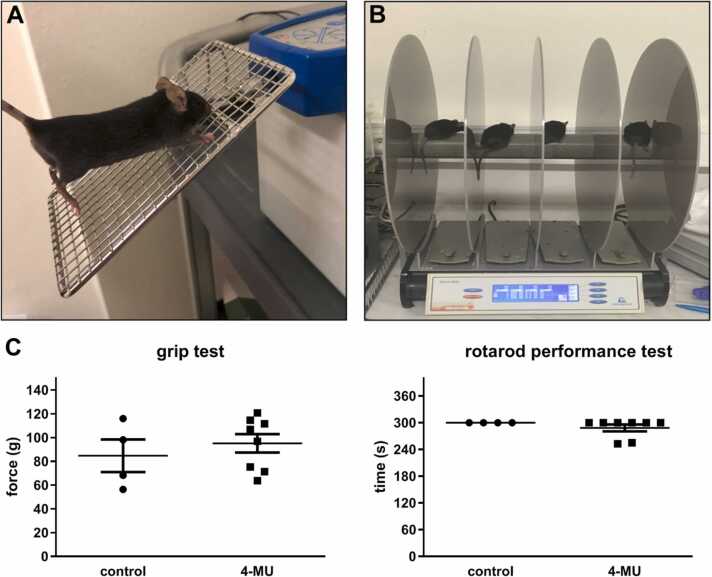
(A) Grip test and (B) rotarod test. (C) The results between control and treated group didn’t show any changes in the strength of the forelimbs period neither motor functions after 6 months of treatment. n = 4 in control and n = 8 in 4-MU group.

In articular cartilage (Fig. 9), we observed an atrophy of the hyaline cartilage with changes in the structure of the cartilage in the 4-MU treated mice, where we observed bone trabeculae with residual, intermittent regenerating hyaline cartilage. However, motor deficit was not observed in rotarod and grip tests (Fig. 10), indicating that the animals were not suffering from joint pain. In both tests, we did not find any differences in balance, coordination, motor-planning and control of the forelimbs between the treated and non-treated group.

In the spleen (Fig. S1A, B), we observed extramedullary haematopoiesis and signs of anaemia in both the control and 4-MU treated groups, which might indicate a compensation for the disrupted haematopoiesis in the bone marrow after 4-MU treatment, described in (Goncharova et al., 2012). We did not observe clear pathological effect of 4-MU in the liver (Fig. S1 C, D). Steatosis as well as extramedullary haematopoiesis was detected in the liver of both control as well as 4-MU treated animals, which was probably due to the high fat in the Western diet (1.25% Cholesterol), which we used to reduce the weight loss of the animals after the 4-MU treatment (Kuipers et al., 2016). No noticeable change of glomeruli and tubules were detected in the kidney from both the control and treated groups (Fig. S2).

Taken together, the histopathological observation from such a high dose of 4-MU is rather mild and suggests a good tolerance. A prolonged systemic 4-MU treatment for 6 months has led to cartilage atrophy which did not affect motor functions. A further study should be performed to systemically interrogate all potential adverse effects of 4-MU in other organs.

## 4 Conclusions

Our study demonstrates that 6-month oral administration of 5% (w/w) 4-MU reduced GAG content in brain, improved memory retention and reduced PNNs in hippocampus in mice after 6 month of treatment. On the other hand, 1 month after the end of the 4-MU treatment, the memory-enhancing effect disappeared while the attenuation of PNNs in the hippocampus continued. Moreover, histopathology revealed mild atrophy of the articular cartilage. Overall, our results provide new knowledge about the effects of systemic inhibition of HA synthesis on the PNNs and suggest that inhibition of HA synthesis might offer a novel therapeutic intervention for PNNs modulation and thus enhancing synaptic plasticity in conditions such as Alzheimer's disease, aging and other disorders of cognition. On the other hand, the detail study of the effects of the systemic inhibition of HA synthesis on synaptic plasticity as well as pharmacological determination of the minimal effective dosage and safety analysis are needed for the future clinically relevant development of 4-MU in modulation of PNNs and their functions. Alternatively, finding non-invasive way to precisely target specific brain area without systemic effects remains a challenge for the future research.

## Declaration of interest

Kwok has a patent ‘Treatment of Conditions of the Nervous System’ (PCT/EP2020/079979) issued.

## References

[bib1] Miyata S., Kitagawa H. (2017). Formation and remodeling of the brain extracellular matrix in neural plasticity: roles of chondroitin sulfate and hyaluronan. Biochim. Biophys. Acta Gen. Subj..

[bib2] Frischknecht R., Heine M., Perrais D., Seidenbecher C.I., Choquet D., Gundelfinger E.D. (2009). Brain extracellular matrix affects AMPA receptor lateral mobility and short-term synaptic plasticity. Nat. Neurosci..

[bib3] Pyka M., Wetzel C., Aguado A., Geissler M., Hatt H., Faissner A. (2011). Chondroitin sulfate proteoglycans regulate astrocyte-dependent synaptogenesis and modulate synaptic activity in primary embryonic hippocampal neurons. Eur. J. Neurosci..

[bib4] Fawcett J.W., Oohashi T., Pizzorusso T. (2019). The roles of perineuronal nets and the perinodal extracellular matrix in neuronal function. Nat. Rev. Neurosci..

[bib5] Sorg B.A., Berretta S., Blacktop J.M., Fawcett J.W., Kitagawa H., Kwok J.C., Miquel M. (2016). Casting a wide net: role of perineuronal nets in neural plasticity. J. Neurosci..

[bib6] Pantazopoulos H., Berretta S. (2016). In sickness and in health: perineuronal nets and synaptic plasticity in psychiatric disorders. Neural Plast..

[bib7] Bozzelli P.L., Alaiyed S., Kim E., Villapol S., Conant K. (2018). Proteolytic remodeling of perineuronal nets: effects on synaptic plasticity and neuronal population dynamics. Neural Plast..

[bib8] Duncan J.A., Foster R., Kwok J.C.F. (2019). The potential of memory enhancement through modulation of perineuronal nets. Br. J. Pharmacol..

[bib9] Romberg C., Yang S., Melani R., Andrews M.R., Horner A.E., Spillantini M.G., Bussey T.J., Fawcett J.W., Pizzorusso T., Saksida L.M. (2013). Depletion of perineuronal nets enhances recognition memory and long-term depression in the perirhinal cortex. J. Neurosci..

[bib10] Kwok J.C., Afshari F., García-Alías G., Fawcett J.W. (2008). Proteoglycans in the central nervous system: plasticity, regeneration and their stimulation with chondroitinase ABC. Restor. Neurol. Neurosci..

[bib11] Howell M.D., Gottschall P.E. (2012). Lectican proteoglycans, their cleaving metalloproteinases, and plasticity in the central nervous system extracellular microenvironment. Neuroscience.

[bib12] Yang S., Hilton S., Alves J.N., Saksida L.M., Bussey T., Matthews R.T., Kitagawa H., Spillantini M.G., Kwok J., Fawcett J.W. (2017). Antibody recognizing 4-sulfated chondroitin sulfate proteoglycans restores memory in tauopathy-induced neurodegeneration. Neurobiol. Aging.

[bib13] Carulli D., Pizzorusso T., Kwok J.C., Putignano E., Poli A., Forostyak S., Andrews M.R., Deepa S.S., Glant T.T., Fawcett J.W. (2010). Animals lacking link protein have attenuated perineuronal nets and persistent plasticity. Brain.

[bib14] Su W., Foster S.C., Xing R., Feistel K., Olsen R.H., Acevedo S.F., Raber J., Sherman L.S. (2017). CD44 transmembrane receptor and hyaluronan regulate adult hippocampal neural stem cell quiescence and differentiation. J. Biol. Chem..

[bib15] Kwok J.C.F., Carulli D., Fawcett J.W. (2010). In vitro modeling of perineuronal nets: hyaluronan synthase and link protein are necessary for their formation and integrity. J. Neurochem.

[bib16] Kakizaki I., Kojima K., Takagaki K., Endo M., Kannagi R., Ito M., Maruo Y., Sato H., Yasuda T., Mita S., Kimata K., Itano N. (2004). A novel mechanism for the inhibition of hyaluronan biosynthesis by 4-methylumbelliferone. J. Biol. Chem..

[bib17] Kultti A., Pasonen-Seppänen S., Jauhiainen M., Rilla K.J., Kärnä R., Pyöriä E., Tammi R.H., Tammi M.I. (2009). 4-Methylumbelliferone inhibits hyaluronan synthesis by depletion of cellular UDP-glucuronic acid and downregulation of hyaluronan synthase 2 and 3. Exp. Cell Res..

[bib18] Vigetti D., Rizzi M., Viola M., Karousou E., Genasetti A., Clerici M., Bartolini B., Hascall V.C., De Luca G., Passi A. (2009). The effects of 4-methylumbelliferone on hyaluronan synthesis, MMP2 activity, proliferation, and motility of human aortic smooth muscle cells. Glycobiology.

[bib19] Ishizuka S., Askew E.B., Ishizuka N., Knudson C.B., Knudson W. (2016). 4-methylumbelliferone diminishes catabolically activated articular chondrocytes and cartilage explants via a mechanism independent of hyaluronan inhibition. J. Biol. Chem..

[bib20] Nagy N., Kuipers H.F., Frymoyer A.R., Ishak H.D., Bollyky J.B., Wight T.N., Bollyky P.L. (2015). 4-methylumbelliferone treatment and hyaluronan inhibition as a therapeutic strategy in inflammation, autoimmunity, and cancer. Front. Immunol..

[bib21] Kuipers H.F., Rieck M., Gurevich I., Nagy N., Butte M.J., Negrin R.S., Wight T.N., Steinman L., Bollyky P.L. (2016). Hyaluronan synthesis is necessary for autoreactive T-cell trafficking, activation, and Th1 polarization. Proc. Natl. Acad. Sci. USA.

[bib22] Mueller A.M., Yoon B.H., Sadiq S.A. (2014). Inhibition of hyaluronan synthesis protects against central nervous system (CNS) autoimmunity and increases CXCL12 expression in the inflamed CNS. J. Biol. Chem..

[bib23] McKallip R.J., Ban H., Uchakina O.N. (2015). Treatment with the hyaluronic Acid synthesis inhibitor 4-methylumbelliferone suppresses LPS-induced lung inflammation. Inflammation.

[bib24] McKallip R.J., Hagele H.F., Uchakina O.N. (2013). Treatment with the hyaluronic acid synthesis inhibitor 4-methylumbelliferone suppresses SEB-induced lung inflammation. Toxins.

[bib25] Yoshioka Y., Kozawa E., Urakawa H., Arai E., Futamura N., Zhuo L., Kimata K., Ishiguro N., Nishida Y. (2013). Suppression of hyaluronan synthesis alleviates inflammatory responses in murine arthritis and in human rheumatoid synovial fibroblasts. Arthritis Rheum..

[bib26] Andreichenko I.N., Tsitrina A.A., Fokin A.V., Gabdulkhakova A.I., Maltsev D.I., Perelman G.S., Bulgakova E.V., Kulikov A.M., Mikaelyan A.S., Kotelevtsev Y.V. (2019). 4-methylumbelliferone prevents liver fibrosis by affecting hyaluronan deposition, FSTL1 expression and cell localization. Int. J. Mol. Sci..

[bib27] Ennaceur A., Delacour J. (1988). A new one-trial test for neurobiological studies of memory in rats. 1: Behavioral data. Behav. Brain Res..

[bib28] Winters J.M., Feng X., Wang Y., Johnson L.M., Foil J. (2004). Progress toward universal interface technologies for telerehabilitation. Conf. Proc. IEEE Eng. Med. Biol. Soc..

[bib29] Bartko S.J., Vendrell I., Saksida L.M., Bussey T.J. (2011). A computer-automated touchscreen paired-associates learning (PAL) task for mice: impairments following administration of scopolamine or dicyclomine and improvements following donepezil. Psychopharmacology.

[bib30] Lin R., Rosahl T.W., Whiting P.J., Fawcett J.W., Kwok J.C. (2011). 6-Sulphated Chondroitins Have a Positive Influence on Axonal Regeneration. PLoS One.

[bib31] Manley G., Hawksworth J. (1966). Diagnosis of Hurler’s syndrome in the hospital laboratory and the determination of its genetic type. Arch. Dis. Child.

[bib32] Trabucchi E., Baratti C., Centemero A., Zuin M., Rizzitelli E., Colombo R. (1986). Controlled-study of the effects of tiropramide on biliary dyskinesia. Pharmatherapeutica.

[bib33] Kuipers H.F., Nagy N., Ruppert S.M., Sunkari V.G., Marshall P.L., Gebe J.A., Ishak H.D., Keswani S.G., Bollyky J., Frymoyer A.R., Wight T.N., Steinman L., Bollyky P.L. (2016). The pharmacokinetics and dosing of oral 4-methylumbelliferone for inhibition of hyaluronan synthesis in mice. Clin. Exp. Immunol..

[bib34] Tsuchiya S., Ohashi Y., Ishizuka S., Ishiguro N., O’Rourke D.P., Knudson C.B., Knudson W. (2020). Suppression of murine osteoarthritis by 4-methylumbelliferone. J. Orthop. Res..

[bib35] Kwok, J.C.F., R. Foster, J.A. Duncan, Treatment of conditions of the nervous system. PCT/EP2020/079979, 2021. 2021-4-29.

[bib36] Baxter M.G. (2010). “I’ve seen it all before”: explaining age-related impairments in object recognition. theoretical comment on Burke et al. (2010). Behav. Neurosci..

[bib37] Yang S., Cacquevel M., Saksida L.M., Bussey T.J., Schneider B.L., Aebischer P., Melani R., Pizzorusso T., Fawcett J.W., Spillantini M.G. (2015). Perineuronal net digestion with chondroitinase restores memory in mice with tau pathology. Exp. Neurol..

[bib38] Kwok J.C., Dick G., Wang D., Fawcett J.W. (2011). Extracellular matrix and perineuronal nets in cns repair. Dev. Neurobiol..

[bib39] Galtrey C.M., Kwok J.C., Carulli D., Rhodes K.E., Fawcett J.W. (2008). Distribution and synthesis of extracellular matrix proteoglycans, hyaluronan, link proteins and tenascin-R in the rat spinal cord. Eur. J. Neurosci..

[bib40] Hylin M.J., Orsi S.A., Moore A.N., Dash P.K. (2013). Disruption of the perineuronal net in the hippocampus or medial prefrontal cortex impairs fear conditioning. Learn Mem..

[bib41] Lewis D.A., Curley A.A., Glausier J.R., Volk D.W. (2012). Cortical parvalbumin interneurons and cognitive dysfunction in schizophrenia. Trends Neurosci..

[bib42] Wen T.H., Binder D.K., Ethell I.M., Razak K.A. (2018). The perineuronal ‘safety’ net? perineuronal net abnormalities in neurological disorders. Front. Mol. Neurosci..

[bib43] Raber J., Olsen R.H., Su W., Foster S., Xing R., Acevedo S.F., Sherman L.S. (2014). CD44 is required for spatial memory retention and sensorimotor functions. Behav. Brain Res..

[bib44] Kwok J.C.F., Carulli D., Fawcett J.W. (2010). In vitro modeling of perineuronal nets: hyaluronan synthase and link protein are necessary for their formation and integrity. J. Neurochem..

[bib45] Rilla K., Oikari S., Jokela T.A., Hyttinen J.M., Kärnä R., Tammi R.H., Tammi M.I. (2013). Hyaluronan synthase 1 (HAS1) requires higher cellular UDP-GlcNAc concentration than HAS2 and HAS3. J. Biol. Chem..

[bib46] Carulli D., Rhodes K.E., Brown D.J., Bonnert T.P., Pollack S.J., Oliver K., Strata P., Fawcett J.W. (2006). Composition of perineuronal nets in the adult rat cerebellum and the cellular origin of their components. J. Comp. Neurol..

[bib47] Arranz A.M., Perkins K.L., Irie F., Lewis D.P., Hrabe J., Xiao F., Itano N., Kimata K., Hrabetova S., Yamaguchi Y. (2014). Hyaluronan deficiency due to <em>Has3</em> knock-out causes altered neuronal activity and seizures via reduction in brain extracellular space. J. Neurosci..

[bib48] Saito T., Dai T., Asano R. (2013). The hyaluronan synthesis inhibitor 4-methylumbelliferone exhibits antitumor effects against mesenchymal-like canine mammary tumor cells. Oncol. Lett..

[bib49] Goncharova V., Serobyan N., Iizuka S., Schraufstatter I., de Ridder A., Povaliy T., Wacker V., Itano N., Kimata K., Orlovskaja I.A., Yamaguchi Y., Khaldoyanidi S. (2012). Hyaluronan expressed by the hematopoietic microenvironment is required for bone marrow hematopoiesis. J. Biol. Chem..

[bib50] Nagy N., Freudenberger T., Melchior-Becker A., Röck K., Ter Braak M., Jastrow H., Kinzig M., Lucke S., Suvorava T., Kojda G., Weber A.A., Sörgel F., Levkau B., Ergün S., Fischer J.W. (2010). Inhibition of hyaluronan synthesis accelerates murine atherosclerosis: novel insights into the role of hyaluronan synthesis. Circulation.

[bib51] Bethesda (MD): National Library of Medicine (US), National Center for Biotechnology Information; 2004–. PubChem Compound Summary for CID 5280567, Hymecromone. PubChem [Internet], 2021.

[bib52] Lontane L. (1968). Toxicological and teratological study of 4-methylumbelliferone. Therapie.

[bib53] Mueller A.M., Yoon B.H., Sadiq S.A. (2014). Inhibition of hyaluronan synthesis protects against central nervous system (CNS) autoimmunity and increases CXCL12 expression in the inflamed CNS. J. Biol. Chem..

[bib54] Sunkari V. (2015). Inhibition of hyaluronan synthesis restores normoglycemia and promotes a regenerative wound phenotype in obese and diabetic mice. Wound Repair Regen..

