# Supplementary material for: Oral treatment of 4-methylumbelliferone reduced perineuronal nets and improved recognition memory in mice
Source: Brain Res Bull. 2022 Apr;181:144–56. doi: 10.1016/j.brainresbull.2022.01.011 (PMC8867078; doi:10.1016/j.brainresbull.2022.01.011)
Supplement: Supplementary file 1 — Supplementary material [file mmc1.docx]

Supplementary material:

**Oral treatment of 4-methylumbelliferone reduce perineuronal nets and improved recognition memory in mice**

Jana Dubisova^a,b^; Jana Svobodova Burianova ^a^; Lucie Svobodova^a,c^; Pavol Makovicky^d^; Noelia Martinez-Varea^a,b^, Anda Cimpean ^a,b^ , James W. Fawcett ^a,e^, Jessica C.F. Kwok ^a,f*^ and Sarka Kubinova^a,g*^

^a^ Institute of Experimental Medicine of the Czech Academy of Sciences, Videnska 1083, 142 20 Prague, Czech Republic

^b^ 2nd Medical Faculty, Charles University, V Úvalu 84, 150 06 Prague, Czech Republic

^c^Institute of Physiology of the Czech Academy of Sciences, Videnska 1083, 142 20 Prague, Czech Republic

^d^ Department of Biology, Faculty of Education, J. Selye University, Slovakia

^e^ John Van Geest Centre for Brain Repair, University of Cambridge, Cambridge, United Kingdom

^f^ School of Biomedical Sciences, Faculty of Biological Sciences, University of Leeds, United Kingdom

^g^ Institute of Physics of the Czech Academy of Sciences, Prague, Czech Republic

**Table S1:** the list of TagMan Gene Expression Assays for quantitative RT-qPCR (Life Technologies, Carlsbad, CA, USA)

| HAS1  Mm03048195_m1 | HAS2  Mm00515089_m1 | HAS3  #Mm00515092_m1 |
| --- | --- | --- |
| HYAL1  Mm00476206_m1 | HYAL2  Mm01230688_g1 | HYAL3  Mm00662097_m1 |
| *ACAN*  *Mm00545794_m1* | *BCAN*  *Mm00476090_m1* | VCAN  #Mm01283063_m1 |
| NCAN  Mm00484007_m1 | NGF  Mm00443039_m1 | GRIA2  Mm00442822_m1 |
| TMEM2  Mm00459599_m1 | TNC  Mm00495662_m1 | SYP  Mm00436850_m1 |
| CD44  Mm01277161_m1 | LYVE  Mm00475056_m1 | SPAM1  Mm00486392_m1 |

**Table S2:** Histopathological evaluation of the control and 4-MU treated animal.

| Cartilage | Normal view (vs. n) | Cartilage Atrophy (vs. n) |
| --- | --- | --- |
| Control (n = 3) | 3/3 | 0/3 |
| 4-MU (n = 4) | 1/4 | 3/4 |

| Spleen | Normal view (vs.n) | Anemia (vs.n) | Extramedullary haematopoiesis (vs.n) |
| --- | --- | --- | --- |
| Control (n=2) | 2/2 | 0/2 | 0/2 |
| 4-MU (n=4) | 0/4 | 1/4 | 3/4 |
| WOE (n=5) | 5/5 | 0/5 | 0/5 |

| Liver | Normal view (vs.n) | Steatosis (vs.n) | Multifocal necrosis (vs.n) | Extramedullary haematopoiesis (vs.n) |
| --- | --- | --- | --- | --- |
| Control (n=4) | 0/4 | 3/4 | 0/4 | 4/4 |
| 4-MU (n=2) | 0/2 | 1/2 | 1/2 | 1/2 |
| WOE (n=5) | 0/5 | 5/5 | 0/5 | 0/5 |


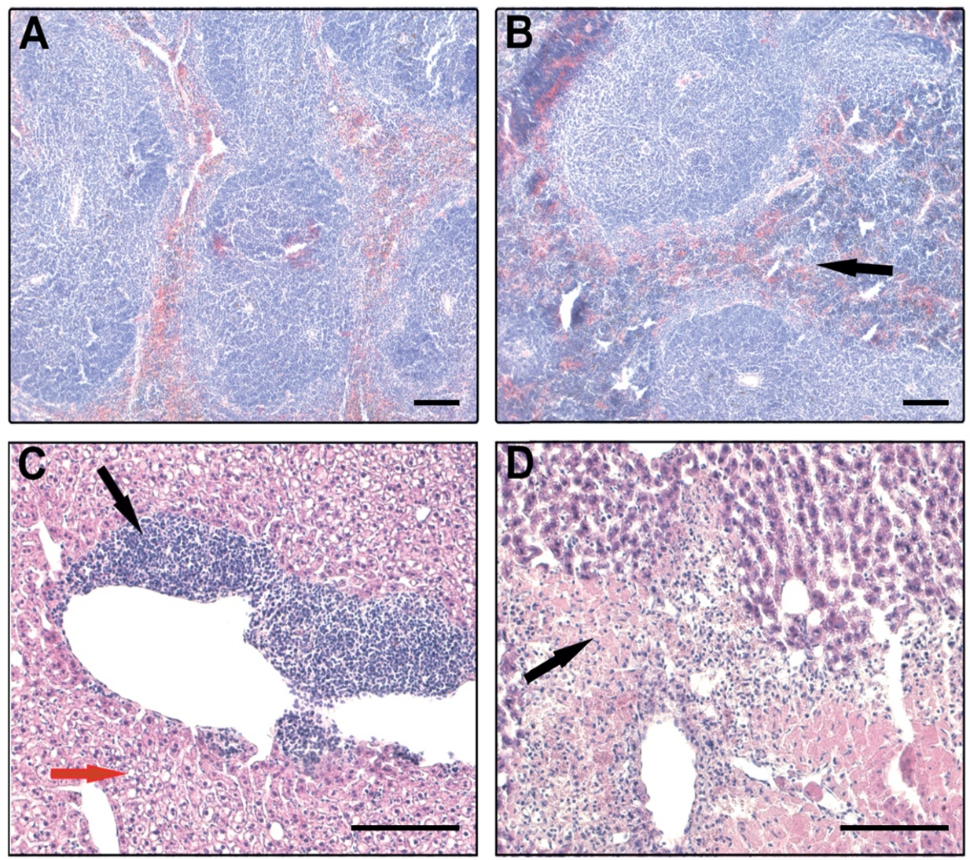


**Figure S1**. Illustrative HE staining of spleen (A, B) and liver (C, D) in control animal (A, C) and after 6 months of 4-MU treatment (B, D). (A) Normal view of spleen in the control animal. (B) Slightly decreased erythrocyte content with signs of extramedullary haematopoiesis in the spleen was observed after 4-MU treatment (black arrow). (C) Simple steatosis (C- red arrow) with extramedullar hematopoiesis (C- black arrow) were detected in the liver in both, the control group and 4-MU group. Liver necrosis with inflammatory reaction (D-black arrow) was observed in 4-MU group. Scale: A,B 100µm, C,D 10µm.

**
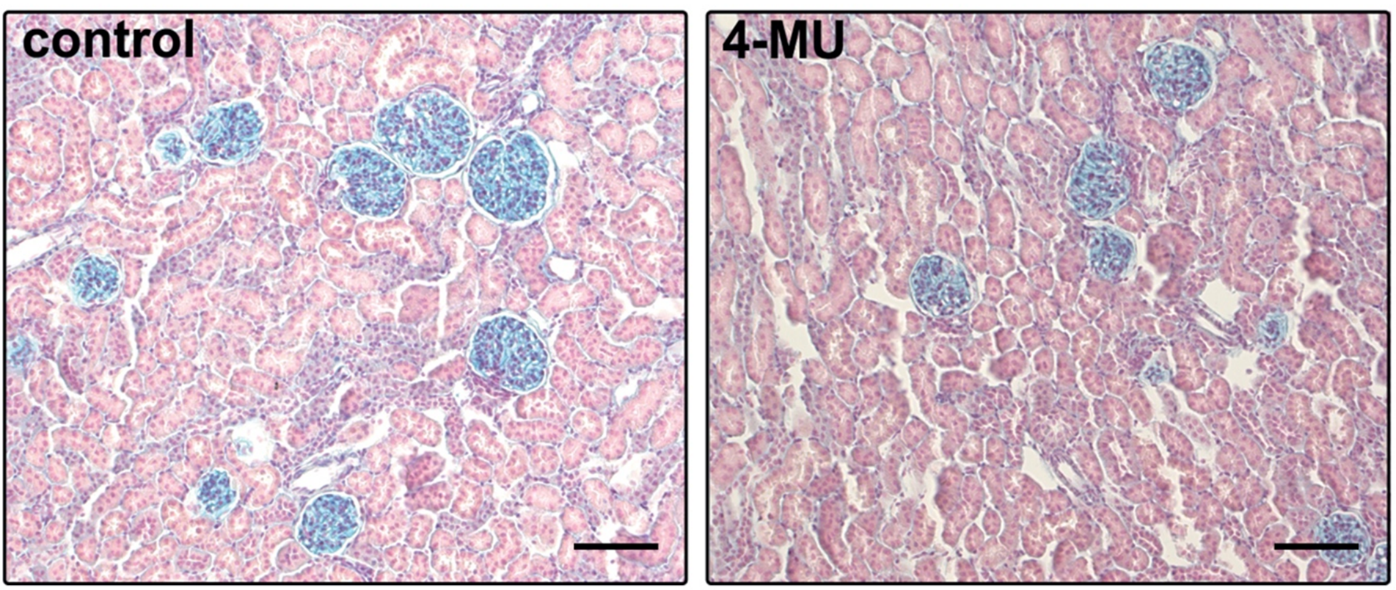
**

**Figure S2.** Illustrative HE staining of kidney in the control animal and after 6 months of 4-MU treatment. No remarkable changes of glomeruli and tubules were detected between control and treated group. Scale bar: 100µm
